# Wearable face mask-attached disposable printed sensor arrays for point-of-need monitoring of alkaline gases in breath

**DOI:** 10.1093/pnasnexus/pgaf116

**Published:** 2025-04-15

**Authors:** Giandrin Barandun, Abdulkadir Sanli, Chun Lin Yap, Alexander Silva Pinto Collins, Max Grell, Michael Kasimatis, Jeremy B Levy, Firat Güder

**Affiliations:** Department of Bioengineering, Imperial College London, London SW7 2AZ, United Kingdom; BlakBear Ltd, 185 Tower Bridge Rd, London SE1 2UF, United Kingdom; Department of Bioengineering, Imperial College London, London SW7 2AZ, United Kingdom; Department of Bioengineering, Imperial College London, London SW7 2AZ, United Kingdom; Department of Bioengineering, Imperial College London, London SW7 2AZ, United Kingdom; Department of Bioengineering, Imperial College London, London SW7 2AZ, United Kingdom; BlakBear Ltd, 185 Tower Bridge Rd, London SE1 2UF, United Kingdom; Department of Bioengineering, Imperial College London, London SW7 2AZ, United Kingdom; BlakBear Ltd, 185 Tower Bridge Rd, London SE1 2UF, United Kingdom; Department of Immunology and Inflammation, Hammersmith Hospital, Imperial College London, London W12 0HS, United Kingdom; Department of Bioengineering, Imperial College London, London SW7 2AZ, United Kingdom

**Keywords:** exhaled breath analysis, face masks, kidney diagnostics, printed paper-based sensor arrays, wearable devices

## Abstract

Blood sampling, despite its historical significance in clinical diagnostics, poses challenges, such as invasiveness, infection risks, and limited temporal fidelity for continuous monitoring. In contrast, exhaled breath offers a noninvasive, pain-free, and continuous sampling method, carrying biochemical information through volatile compounds like ammonia (NH_3_). NH_3_ in exhaled breath, influenced by kidney function, emerges as a promising biomarker for renal health assessment, particularly in resource-limited settings lacking extensive healthcare infrastructure. Current analytical methods for breath NH_3_, though effective, often face practical limitations. In this work, we introduce a low-cost, internet-connected, paper-based wearable device for measuring exhaled NH_3_, designed for early detection of kidney dysfunction at the point of need. The device, which attaches to disposable face masks, utilizes an array of disposable paper-based sensors to detect NH_3_ with the readout being changes in electrical impedance that correlate with the concentration of NH_3_. The sensor array is housed in a biodegradable plastic enclosure to mitigate high relative humidity issues in breath analysis. We validated our technology using a laboratory setup and human subjects who consumed ammonium chloride-containing candy to simulate elevated breath NH_3_. Our wearable sensor offers a promising solution for rapid, point-of-need kidney dysfunction screening, particularly valuable in resource-limited settings. This approach has potential applications beyond kidney health monitoring, including chemical industry safety and environmental sensing, paving the way for accessible, continuous health monitoring.

**Significance Statement**

This research presents a low-cost, wearable device that enables continuous, noninvasive monitoring of ammonia in exhaled breath—a promising biomarker for kidney dysfunction. Unlike traditional blood tests, which are invasive and challenging in resource-limited settings, our device offers a simple and real-time alternative for early detection of kidney problems, particularly valuable in areas with limited healthcare infrastructure. The device is integrated with a disposable paper-based sensor array, housed in a biodegradable enclosure, and can be easily attached to a face mask for use in various environments. Beyond kidney health, this technology has the potential to monitor a range of volatile compounds, expanding its applications to fields like environmental monitoring, industry safety, and food quality control.

## Introduction

Blood has historically been the sample matrix of choice when searching for or measuring biochemical markers of health and disease in the body (1). Accessing blood for biochemical analysis, however, is challenging for at least three reasons: (i) Blood samples require painful procedures for extraction (this is, especially problematic in children); (ii) the risk of infection is increased when the skin barrier is damaged; and (iii) for an extensive range of measurements, the amount of blood needed limits the frequency of analysis, therefore reducing temporal fidelity that requires continuous-time measurements, reducing diagnostic performance.

The respiratory rate of a healthy human is between 12 and 18 breaths times per minute (2). Unlike blood, breathing provides easy access to a gaseous sample matrix (that is, exhaled breath) carrying information concerning the internal biochemistry of the body through the exchange of gases in the respiratory tract, including the mouth (3). Breath-based, noninvasive, pain-free assessments can be performed continuously over time with a high frequency of measurement, which is not easily achievable through blood-based measurements with implants or skin-attached microneedles (4).

Human breath contains a range of volatile organic and inorganic compounds, including biomarkers such as isoprene, ammonia (NH_3_), and acetone, which can be used to predict various disease states (5–7). NH_3_ is a toxic byproduct of protein metabolism, which is produced microbially in the gut and cellularly throughout the body (8). NH_3_ and its ionized form ${\mathrm{NH}}_{4}^{+}$ are removed from the body either directly or by conversion into urea (CH₄N₂O) in the liver through the urea cycle (9). Urea is a highly water-soluble, practically nontoxic, small molecule that is stable in the body (10). Urea can be rapidly hydrolyzed into CO_2_ and NH_3_ catalytically in the presence of urease, an enzyme that is only produced microbially; hence, urease is not an endogenous enzyme (10). Because of its small size, urea diffuses readily into tissues, including the oral cavity and saliva (10). When kidneys function abnormally, urea concentrations in the blood and tissues increase; therefore, blood urea nitrogen (BUN) concentration is used as a diagnostic biomarker to assess kidney health (11). In the oral cavity, urea is hydrolyzed by the urease-positive microbes into NH_3_, which is expelled from the body with exhaled breath (12).

Patients suffering from end-stage kidney failure from any cause exhale higher NH_3_ levels in their breaths (820–14,700 ppb, with a mean of 4,880 ppb) compared with healthy individuals (425–1,800 ppb, mean of 960 ppb) (13). Kidney dysfunction, caused by chronic kidney disease (CKD) or acute kidney injury (AKI), is currently diagnosed by measuring serum creatinine (sCreat) and BUN, both blood-based tests (14). Assessing kidney health noninvasively, rapidly, and at a low cost is especially important in resource-limited settings (RLSs) where blood testing is not widely available, such as in low- and middle-income countries (15). In developing nations where healthcare is severely lacking outside major centers, AKI that goes undiagnosed due to lack of diagnostic testing leads to preventable deaths, and CKD can only be diagnosed currently by blood testing, causes no symptoms, is an increasing worldwide public health issue, and is potentially treatable (16). Exhaled NH_3_ is, therefore, particularly suited for use as a diagnostic marker to measure kidney (dys)function in RLS to prevent premature deaths due to CKD and AKI; an estimated 13.3 million cases of AKI occur in developing nations annually (17).

To avoid blood-based testing, to date, analytical techniques, such as gas chromatography/mass spectrometry (GC–MS), selected-ion flow-tube MS (SIFT-MS), laser spectroscopy, and laser photoacoustic spectroscopy, have been developed for measuring breath NH_3_ (9, 18–21). These methods are, however, bulky, costly, and impractical for use in RLS as a diagnostic technique due to preanalytical errors and challenges associated with sample handling. Other approaches, such as quartz crystal microbalance (22), chemical (23), and optical sensors (24), can perform with high precision at trace levels, but they face limitations in terms of analytical performance. For most sensing technologies, the high content of moisture in breath (human breath is >90% in relative humidity [RH]) creates a myriad of analytical issues (25). Moisture can poison catalytic surfaces and block sensing surfaces by adsorption or condensing into liquid droplets, which in turn damages sensors or electronics (26). The presence of a high content of water in breath, therefore, limits the analytical performance of most low-cost sensing approaches. High RH of exhaled breath has been the primary factor preventing the development of breath-based diagnostics (27, 28).

**Fig. 1.**
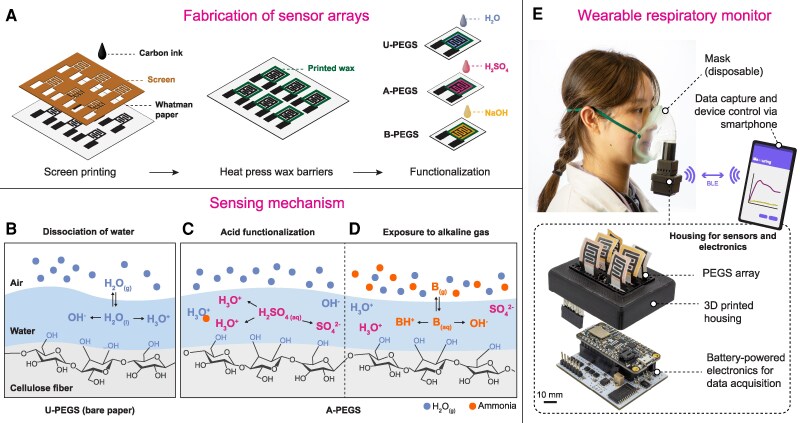
Fabrication of sensor arrays. A) Illustration of carbon ink electrodes on chromatography paper with wax squares for liquid confinement. B) Mechanism of conductivity in the paper due to water accumulation around cellulose fibers with increasing RH. C) Functionalization process involving sulfuric acid (H_2_SO_4_) on paper electrodes to modify electrical impedance. D) Response of acid-treated sensors to alkaline gases, such as NH_3_, affecting electrical impedance. E) Application of sensor arrays in a wearable respiratory monitor attached to a medical mask, with BLE connectivity for data transmission. Illustrations not to scale.

In this work, we report a low-cost, internet-connected, paper-based wearable device for measuring exhaled NH_3_ in human breath, with the goal of producing a noninvasive method for early detection of kidney dysfunction at the point of need (Fig. 1). The technology reported consists of a disposable paper-based sensor array to overcome RH-related artifacts, housed in a biodegradable plastic enclosure. The device attaches to disposable face masks commonly used in healthcare. The test results are transmitted to a nearby smartphone for postprocessing and can be shared with a remote professional over the internet; hence, they are compatible with telemedicine (29). We validated our approach through NH_3_ using a characterization environment in our laboratory and human subjects, who consumed a candy (salty licorice) that contains NH_4_Cl to generate NH_3_ in exhaled breath.

## Results and discussion

### Fabrication of sensors

Paper-based gas sensors (PEGSs) are produced by screen printing carbon electrodes onto Whatman chromatography paper, allowing rapid prototyping and low-cost ($0.02/sensor; see Table S1) production of PEGS (Fig. 1A). For selectively detecting NH_3_ in exhaled breath, we used an array of sensors that consisted of three types of functionalization: (i) untreated-PEGS (U-PEGS), produced by adding 10 µL of deionized (DI) water; (ii) base-treated PEGS (B-PEGS), produced by adding 10 µL of sodium hydroxide (0.001–0.1 M NaOH); and (iii) acid-treated PEGS (A-PEGS), produced by adding 10 µL of sulfuric acid (0.001–0.1 M H_2_SO_4_) to the paper. Wax barriers created around the carbon electrodes prevented the spread of the solutions of acid or base, spatially confining the functionalized regions of the paper. Because the chemical compounds added slowly react with environmental gases such as CO_2_, the functionalization was performed right before the start of the experiments, no longer than 15 min before the sensor testing. To preserve their functionality, PEGS should be stored in sterile, gas-impermeable packaging to prevent premature exposure to environmental gases, ensuring accurate and reliable readings in real-world applications. Notably, NH_3_ has the highest concentration in exhaled breath among alkaline gases, and PEGS produces the highest response to NH_3_ among all gases tested (30). Therefore, in this work, NH_3_ is the primary alkaline gas we are sensing.

### Mechanism of sensing

Paper is a highly hygroscopic material that consists of natural microfibers of cellulose, which absorb moisture from the immediate environment. At higher levels of RH (>40%), the water present around the fibers of cellulose within paper behaves like bulk water (31). Ions in the layer of water adsorbed in the paper can move freely through the network of cellulose fibers, rendering the paper electrically more conductive (Fig. 1B) (30). A water-soluble gas that dissolves and dissociates into ions in water can, therefore, increase the number of ions in paper, leading to higher electrical conductivity (30). The electrical conductivity (*σ*) of water depends on the concentration (*n*_ion_), charge (*Z*_e_), and mobility (*µ*_ion_) of ions present such that *σ* = *n*_ion_ × *Z*_e_ × *µ*_ion_. When paper is pretreated with H_2_SO_4_, two additional hydronium ions (2H_3_O^+^) and one sulfate ion (${\mathrm{SO}}_{4}^{2-}$) are produced for each H_2_SO_4_ molecule dissolved in water (Fig. 1C), leading to higher electrical conductivity. When, however, an alkaline gas, such as NH_3_, reacts with the sulfuric acid functionalized paper (A-PEGS), NH_3_ dissolves in the water layer on the cellulose fibers and dissociates to form water (H_2_O), hydroxide ions (OH⁻), and ammonium ions (${\mathrm{NH}}_{4}^{+}$). The hydroxide ions (OH⁻) neutralize the acidity from the sulfuric acid, resulting in the formation of water (H_2_O) and ammonium sulfate ([NH_4_]_2_SO_4_). [NH_4_]_2_SO_4_ is a highly water-soluble salt and will be present in its dissociated form (two ammonium ions [2${\mathrm{NH}}_{4}^{+}$] and one sulfate ion [${\mathrm{SO}}_{4}^{2-}$]) when dissolved. The addition of NH_3_ to aqueous sulfuric acid substitutes hydronium ions (H_3_O^+^) with ammonium ions (${\mathrm{NH}}_{4}^{+}$; Fig. 1D). Because ${\mathrm{NH}}_{4}^{+}$ ions have a lower mobility than H_3_O^+^ (7.63 × 10^−4^ vs. 36.23 × 10^−4^ cm^2^ V^−1^ s^−1^), the reaction of NH_3_ with A-PEGS functionalized with H_2_SO_4_ causes a drop in the electrical conductivity of paper (32). The mechanism of sensing acidic gases with B-PEGS is similar to A-PEGS, which increases selectivity toward the detection of acidic gases.

The electrical conductivity of paper changes both when it reacts with a water-soluble gas and when the RH increases. A single PEGS (with or without chemical modifications) would, therefore, have low selectivity when operating in a multicomponent mixture of gas. To increase selectivity toward a target acidic or alkaline gas (i.e. NH_3_) in the presence of fluctuating levels of RH, we used an array of sensors consisting of U-PEGS, A-PEGS, and B-PEGS. The responses produced by each sensor within the array could then be used for differential analysis to calculate the concentration of the target gas, as different sensors would react with the target gas to a varying extent. Integrated into a disposable face mask, the array of sensors would allow noninvasive monitoring of levels of exhaled breath NH_3_ where the RH would be changing in each cycle of inhalation and exhalation (Fig. 1E).

### Characterization

**Fig. 2.**
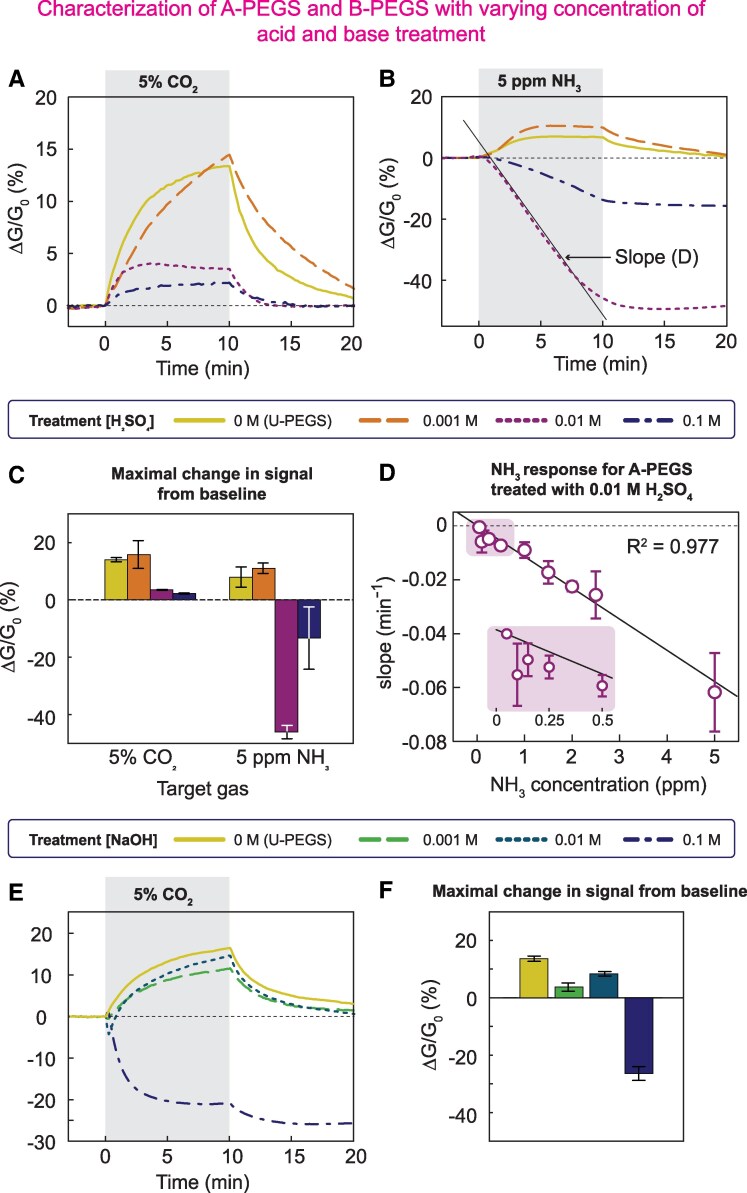
Characterization of A-PEGS and B-PEGS with varying concentrations of acid and base treatment in the test chamber: the conductance of PEGS is measured when a sinusoidal signal of 4 V, and 10 Hz is applied to the sensors (see SI-P3 for details). A) Four PEGS were treated differently: one U-PEGS and three A-PEGSs with different concentrations of H_2_SO_4_. All PEGSs were exposed to 5% CO_2_ for 10 min. The bar plot shows maximal signal changes in a 10-min interval for the differently treated sensors, and the error bars indicate the SD for *n* = 3. B) The same array used in (A) is exposed to 5 ppm of NH_3_ for 10 min. For high acid concentrations (0.01 and 0.1 M), the alkaline gas neutralizes the acidic pretreatment, and the signal drops constantly over time (slope is the black solid line). For the lowest concentration (0.001 M), the acid is depleted quickly, and the sensor starts behaving like a U-PEGS. C) Maximal change in signal from baseline for the A-PEGS exposed to 5% CO_2_ and 5 ppm of NH_3_. D) A-PEGS treated with 0.01 M H_2_SO_4_ shows the best signal in terms of error and especially sensitivity to NH_3_ (see (C)). We exposed these A-PEGS to a wide range of NH_3_ concentrations (0.05 to 5 ppm) and calculated the slope of the drop (black solid line in (B)). This gives a linear correlation between slope and NH_3_ concentration with a coefficient of determination of *R*^2^ = 0.977. At lower concentrations (pink inlet), the errors get bigger. B-PEGS characterization with varying concentrations of alkaline treatment. (E) Four PEGSs were treated differently: one U-PEGS and three B-PEGSs with different concentrations of NaOH. One bare-PEGS is compared with three B-PEGSs when exposed to 5% CO_2_ for 10 min. F) The acidic gas neutralizes the alkaline pretreatment and the signal for B-PEGS initially drops. For low concentrations of base (0.001 and 0.01 M), the PEGSs are depleted quickly, and the signal starts increasing again. The error bars are the SD for *n* = 3–12.

To study the behavior arrays of sensors consisting of U-PEGS and A-PEGS and U-PEGS and B-PEGS, we exposed the arrays to different concentrations of NH_3_ and CO_2_ (relevant gases for the analysis of levels of NH_3_ in exhaled breath) while keeping the RH at 65% (Fig. 2). Fixing the RH constant enabled us to characterize the behavior of each sensor (array) toward the target analytes in a more precise fashion, which is important for understanding the underlying phenomena. To account for the intrinsic microstructural variability of paper and chemical modifications, the electrical responses originating from each sensor were normalized to the baseline conductance—i.e. Δ*G*/*G*_0_ (*G*_0_ is the electrical conductance of PEGS in the absence of the target gas; Δ*G* is the change in electrical conductance when exposed to the target gas).

When exposed to 5% CO_2_, A-PEGS (treated with 10 µL of 0.001 to 0.1 M H_2_SO_4_) shows a reversible response (Fig. 2A). We observed that increased concentration of H_2_SO_4_ decreases the sensitivity of CO_2_ in two ways: (i) the base conductance (*G*_0_) is higher with sensors containing higher amounts of H_2_SO_4_ and, therefore, the relative change (Δ*G*) is smaller; and (ii) decreasing pH reduces the solubility of CO_2_ in water, leading to lower increases in net ionic strength, hence lower sensitivity (33).

When the array was exposed to 5 ppm NH_3_, we observed two different behaviors (Fig. 2B). For the lowest concentration of H_2_SO_4_ (0.001 M, orange, long dashed), A-PEGSs behave like U-PEGS and show an increase in response. For higher concentrations of added sulfuric acid (0.01 and 0.1 M), we observed a continuous drop in signal. This behavior can be explained by the ongoing neutralization of the H_2_SO_4_ by the dissolved NH_3_ in the paper. When the concentration of H_2_SO_4_ was small, initial H_2_SO_4_ was immediately neutralized without much effect on the overall electrical conductance. When the concentration of the acid increased, exposure to NH_3_ produced a noticeable drop in the response of the A-PEGS. The rate of the change of conductance over time (slope) could, therefore, be used as an indicator of the NH_3_ concentration (e.g. straight gray line in Fig. 2B for 0.01 M H_2_SO_4_). Comparing the treatment with 0.01 M H_2_SO_4_ to 0.1 M (Fig. 2C), we found that the sensitivity to 5 ppm NH_3_ is approximately three times higher, and the SD is five times smaller (*n* = 3) for A-PEGS treated with 0.01 M H_2_SO_4_. The increased variability in the response for the devices modified with 0.1 M H_2_SO_4_ is likely due to pipetting errors since a slight increase or decrease in the dispensed volume would introduce substantially more sulfuric acid to the device, leading to larger variations in the slope. Since A-PEGS with 0.1 M H_2_SO_4_ modification is not used in human experiments, however, the variability is of no concern to the performance of the sensor arrays in detecting exhaled NH_3_.

Figure 2D shows the relative rates of change of conductance (min^−1^, straight gray line Fig. 2B) for A-PEGS treated with 0.01 M H_2_SO_4_ when exposed to concentrations of NH_3_ ranging from 0.05 to 5 ppm (Fig. S1). We observed a linear correlation between the slope of the response of A-PEGS and NH_3_ concentration (*R*^2^ = 0.977) as exposure to higher levels of NH_3_ neutralizes the acid more quickly. For lower concentrations (0.05–0.25 ppm), the data exhibited higher SD and lower linear correlation ($R_{0.05\text{–}0.25\mathrm{ppm}}^{2}$ = −0.95). Longer exposure times improve the limit of quantification because the increased total amount of NH_3_ passing the sensors enhances detection sensitivity.

For B-PEGS, the response to CO_2_ is more complicated (Fig. 2E). For 0.1 M NaOH (purple, dot-dashed), we see a drop in conductance as the base gets neutralized by the acidic gas. After gas exposure is stopped, we see another drop in conductance because additional bicarbonate ions (${\mathrm{HCO}}_{3}^{-}$) form CO_2_, which is released from the sensor into the environment again. For a lower base concentration (0.01 M NaOH, green, long dashed), the base is neutralized within the first minute (sharp drop in conductance). After that, bicarbonate ions form in the sensing element, which increases conductance. For the lowest base concentration (0.001 M NaOH, cyan, dashed), we do not see a neutralization effect. The base gets neutralized by the acidic gas within a few seconds, and the sensor shows a conductance increase due to bicarbonate ions forming in the sensing element.

### Detecting exhaled NH_3_ in a respiratory simulator

In each cycle of breathing, the RH immediately outside the oronasal opening fluctuates between 100% RH and room RH (inhalation). To characterize the performance of the PEGS array in the presence of fluctuating levels of RH and NH_3_, we built a respiratory simulator (Fig. S2). The simulator cycles the RH in the gas sensor characterization chamber between 100 and 45% RH (the RH in our laboratory) at a (adjustable) frequency of six breaths per minute while introducing a controlled concentration of NH_3_ in each cycle of exhalation.

**Fig. 3.**
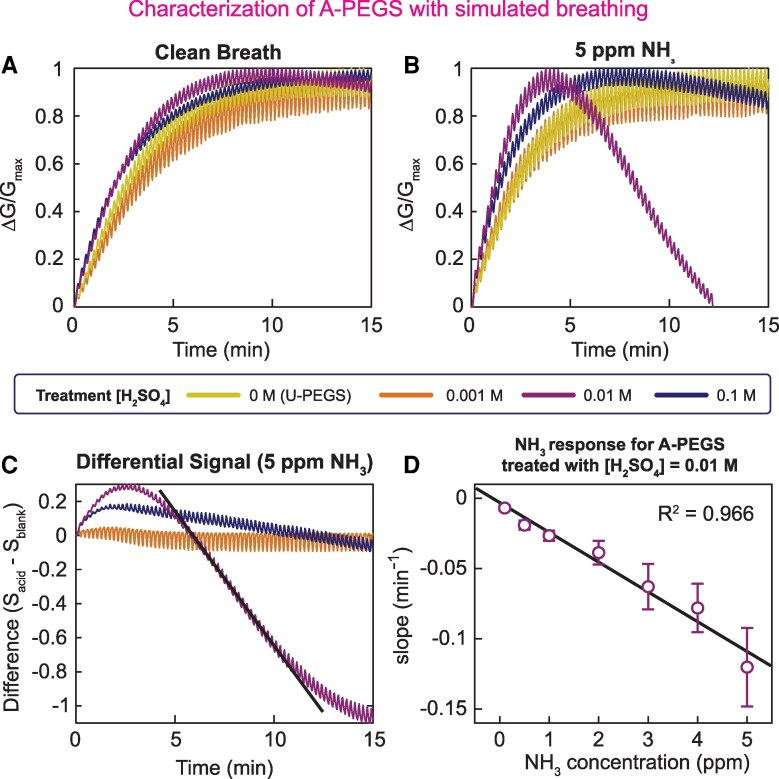
Characterization of A-PEGS with simulated breathing: we tested PEGS arrays consisting of U-PEGS and A-PEGS in our respiratory simulation chamber. Breathing was simulated by exposing the PEGS in turns to dry air (ca. 50% RH) and humidified air (ca. 90% RH). We mixed different concentrations of NH_3_ (0.1–5 ppm) into the compressed air to simulate NH_3_ in breath. A) The response of the array to clean breath (no NH_3_). The signal rises until the sensors reach an equilibrium with the breathing. We normalized the signal for better comparison and all sensors behave similarly. B) We exposed the array to breath containing 5 ppm NH_3_. The U-PEGS and lowest acid concentration A-PEGS (0.001 M) show no change to clean breath. We see a decline after the initial peak for the A-PEGS treated with higher concentrations of H_2_SO_4_ (0.01 and 0.1 M). Similar to the previous tests, the A-PEGS with 0.01 M H_2_SO_4_ shows the highest sensitivity, and the decline can be seen clearly. C) To decouple the signal from any environmental influence (especially RH), we take the difference in the signal of the U-PEGS and the A-PEGS (S_A-PEGS_ − S_U-PEGS_). The slope of the differential signal after the initial peak (dashed line) can now be used to determine the NH_3_ concentration. D) We plotted the slope (straight black line in (C)) for A-PEGS treated with 0.01 M H_2_SO_4_ against different concentrations of NH_3_ in breath ranging from 0.1 to 5 ppm. The data show a linear correlation (*R*^2^ = 0.966). The error bars indicate the SD for *n* = 3.

We first subjected a PEGS array consisting of three A-PEGSs and three U-PEGSs to simulated cycles of respiratory activity without any NH_3_ (Fig. 3A). The desorption of moisture from paper is thermodynamically less favorable than adsorption (34). Simulated cycling of respiratory activity, therefore, slowly increases the moisture content within the paper, eventually reaching a steady state after 7–8 min regardless of the amount of acid added to the paper in the context of A-PEGS. Next, we introduced 5 ppm of NH_3_ along with respiratory cycling (Fig. 3B). Although NH_3_ started neutralizing H_2_SO_4_ present in A-PEGS immediately, we first observed a steady increase in the response of the sensor. After about 4 min, the A-PEGS with 0.01 M H_2_SO_4_ exhibited a steady drop in response as expected. The initial increase and subsequent decrease in the response of the sensor can be attributed to two competing processes moisture build-up vs. neutralization of H_2_SO_4_ in which the latter eventually dominates the overall electrical conductance. All the other sensors showed primarily an increase in the response of the sensor since the adsorption of moisture dominated the electrical signal.

We exploited the dominance of the RH response as opposed to neutralization in the non- or slightly modified sensors in the array for differential analysis (Fig. 3C). Differential analysis yields a curve in which the effect of moisture is subtracted from NH_3_ thereby allowing calculation of the drop, hence NH_3_ concentration in exhaled (simulated) breath. Mathematically, the isolation of the alkaline gases, such as the NH_3_ signal is achieved by subtracting the signal of the U-PEGS, which primarily responds to RH changes from the signal of the A-PEGS, which responds to both NH_3_ and RH. Here, the equation used is $S_{NH_{3}}$ = *S*_A-PEGS_ − *S*_U-PEGS_, where *S*_U-PEGS_ is the total response of the A-PEGS, and S_U-PEGS_ is the response of U-PEGS to RH. The slope of the differential signal measured at varying levels of NH_3_ produced a linear relationship (*R*^2^ = 0.966) with a limit of detection (LOD) is 0.1 ppm of NH_3_ after 15 min (Fig. 3D) simulated respiratory cycling. To achieve lower LOD than 0.1 ppm, a longer duration of respiratory cycling may be necessary.

### Human testing

**Fig. 4.**
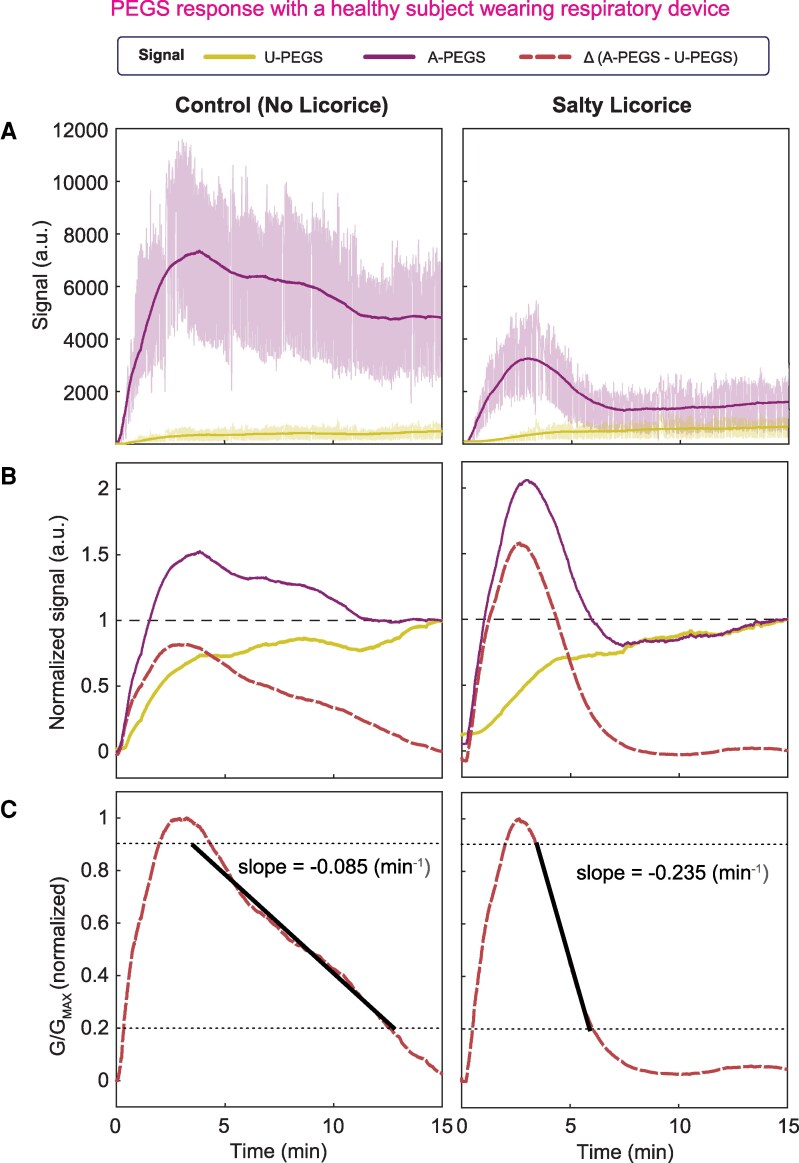
PEGS response with a healthy subject wearing respiratory device. A) The raw signal obtained from a human subject breathing into the face mask, therefore, on the sensors, for 15 min, is shown in purple for A-PEGS and yellow for U-PEGS. To eliminate the signal fluctuations due to inhalation and exhalation, we use a 1,000-point moving average (pink line). This can track the conductivity changes in the PEGS from humidity and ion changes in the breath without the fluctuations of breathing. The human subjects were asked to do a control experiment (normal breathing, left), and an experiment where they were eating a salty licorice while doing the respiratory experiment (right). B) We divide the moving average from (A) by the endpoint after 15 min to relate the signal from U-PEGS and A-PEGS. Then, we subtract the two signals to filter the changes from an increased RH. This difference tracks the drop of the A-PEGS due to the neutralization of the sulfuric acid by alkaline gases (i.e. NH_3_). C) We normalized the difference from (B) to compare different subjects and experiments. The slope is calculated on an interval from 90% peak height to 20%. The straight gray dashed lines show the interval we use to calculate the slope of the drop. Control experiments with no salty licorice (left) show a flatter decrease than experiments with salty licorice (right).

In a series of experiments, we tested our approach for measuring levels of NH_3_ in exhaled breath, we produced a wireless sensor module (see SI-P3) that can be attached to a disposable face mask for wearable, point-of-care analysis of breath (Fig. 1E). The PEGS array used in these experiments comprised three A-PEGSs treated with 0.01 M H_2_SO_4_ and three U-PEGSs. We tested our respiratory device with eight healthy male volunteers from our research group (body mass index 18–26, age 22–34). Because all subjects participating in the study were healthy, to simulate kidney disease, we asked the volunteers to consume salty licorice candy (Malaco Salmiak Balk Sweet from *Scandinavian Candy & Sweets*) which contains copious amounts of NH_4_Cl. The human experiments (Fig. 4) consisted of two parts: (i) we first asked the volunteers to wear the respiratory monitor for 15 min and breathe normally through their mouths; and (ii) we then gave each volunteer a piece of candy and once again asked them to wear and breathe through the mask while consuming the candy without chewing.

For differential analysis, we first applied a 1,000-point averaging low-pass filter to smoothen the signals acquired from the sensors (Fig. 4A), as the raw measurements were noisy. Next, we normalized each signal to the final data point acquired at the 15-min mark (Fig. 4B) to allow subtraction of the response generated by U-PEGS from A-PEGS to produce a differential signal. We finally calculated the slope of the differential signal in the linear region before the signal flatlined, which would indicate the completion of the neutralization reaction between exhaled NH_3_ and H_2_SO_4_ present in the paper.

**Fig. 5.**
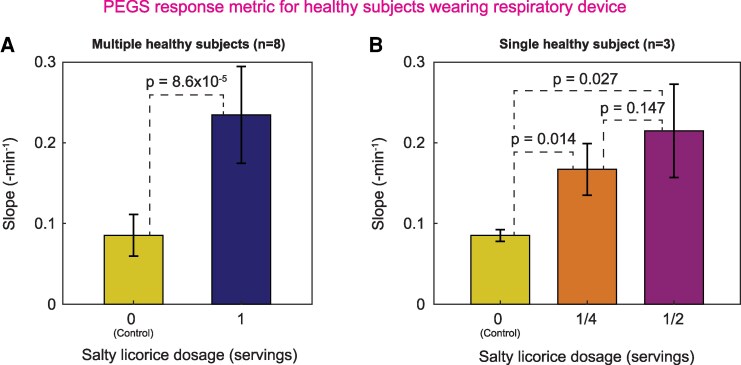
A) The results of the mean slope (see Fig. 4C) for eight healthy subjects in an experiment with normal breathing (control) and breathing while eating a salty licorice. The slope increases 3-fold when the NH_3_ from the candy neutralizes the sulfuric acid in the A-PEGS. B) A similar experiment to (A) with one subject only and three repeats on each bar. In addition to the control experiment, the subject was asked to eat ¼ and ½ of a salty licorice during the 15-min experiment. The error bars indicate the SD for (A) *n* = 8 and (B) *n* = 3. The *P*-values come from one-tailed (right) paired samples t test, indicating a significant difference if *P* < 0.05.

Our tests (Fig. 5A) with the healthy volunteers (*n* = 8) showed a statistically significant difference (paired t test; *P* < 0.05) between the healthy and control groups (i.e. simulated diseased state) when the subjects consumed either no candy or a single full candy. The slope in the control experiment might come from small amounts of NH_3_ that can be present in healthy humans (35). Even though the amount of NH_3_ might vary from subject to subject, the concentration in healthy human breath is negligible compared with the concentration in breath following the consumption of salty licorice. We conducted another experiment with a single volunteer who was asked to consume smaller quantities of the candy (*n* = 3): ½ and ¼ of a single candy (Fig. 5B). Although there was a clear difference between the average slopes calculated for the control (no candy), ½, and ¼ of a single candy experiment, increasing the candy amount from ¼ to ½ does not make a significant difference according to the statistical tests (one-tailed t test).

## Conclusion

The sensing technology reported is a low-cost approach to analyze the chemical composition of exhaled breath across a large range of concentrations (i.e. high dynamic range) without depending on collecting a breath condensate, which is the most common method of analyzing exhaled breath (36, 37) (Tables 1 and S3). By not depending on collecting condensation, it is possible to rapidly detect chemical compounds with simpler instrumentation. For our laboratory prototypes, each sensor cost ∼US $0.02 to produce, which would need to be replaced before each measurement. The electronics and plastic housing cost US $75 (Tables S1 and S2); however, these components are reusable after disinfection. All the components of the plastic housing were made of biodegradable polymers and can also be disposed of which would, of course, slightly raise the unit price of each test. Although in this work, the plastic components were 3D printed, they can also be injection molded; hence, every single element within our design is compatible with the existing high-volume manufacturing methods except for wax printing, which would need to be replaced with an appropriate alternative. There is, therefore, a large scope for reducing the cost of the device proposed, potentially one to two orders of magnitude at large scales of production.

**Table 1.** Recent advances in NH_3_ gas sensors for breath analysis.

Technology/Material	Humidity	LOD (ppb)	Real-time^a^	Year (Ref.)
PANI nanojunction	Dried	16	Sample bag	2008 (38)
MoO_3_	Controlled^b^	50	Simulation	2010 (39)
DFB-QCL^c^	Real breath	6	Yes	2011 (18)
PANI nanoparticles	Real breath	40	Yes	2013 (40)
QCM^d^ (SiO_2_)	Constant	1,000	Samples	2015 (22)
Si-doped α-MoO_3_	Constant (90%)	400	No	2015 (41)
CuBr	Constant (40%)	10	Yes^e^	2016 (42)
ssDNA-FG^f^	Constant (80%)	103	No	2017 (43)
TFB^g^	Dried (10%)	<100	Sample bag	2017 (44)
IL-SOWG^h^	Dried	69	Sample bag	2018 (24)
D-A^i^ polymer nanopores	Dried (10%)	100	Sample bag	2019 (45)
PVP^j^	Constant (97%)	500	Sample chamber	2019 (46)
Au NP^k^-V_2_O_5_/CuWO_4_	Constant (n.a.)	212	No	2020 (47)
CuBr film	Dried^l^	100	Yes	2020 (38)
BaFe_12_O_19_ NP^k^	Dried	200	No	2020 (48)
PEGS array (this work)	Real breath	100	Yes	2025

^a^A device tested in real-time on a human subject.

^b^Humidity controllable test chamber but not mentioned at what RH experiments are conducted.

^c^DFB-QCL: distributed feedback quantum cascade laser (not handheld).

^d^QCM: quartz crystal microbalance sensor.

^e^Additional equipment to control the humidity is needed for real-time breath analysis.

^f^ssDNA-FG: single-stranded DNA-functionalized graphene.

^g^TFB: (poly[(9,9-dioctyl-fluor-enyl-2,7-diyl)-co-(4,4′-(*N*-(4-s-butylphenyl)diphenylamine)]).

^h^IL-SOWG: ionic liquid-based slab optical waveguide sensor.

^i^D-A: electron donating and electron accepting.

^j^PVP: poly(vinyl pyrrolidone), impedimetric.

^k^NP: nanoparticles.

^l^Exhaled breath was passed over a quicklime bag and additionally, corrections to the signal due to humidity changes were calculated (humidity sensor needed).

The sensing platform reported, however, has at least four disadvantages. (i) The screen-printed graphite electrodes are susceptible to cracking if the paper is creased, but this is unlikely during use as the sensors were securely placed inside the respiratory device without folds. (ii) Performance of PEGS is hindered by the lower levels of RH (<20%) and temperatures below 0 °C due to the freezing point of water. Additionally, because paper is an organic substrate, PEGSs are unsuitable for operation at high temperatures (>∼120 °C) though such conditions are rare for biological applications. (iii) The impedance of PEGS ranges from kΩ to GΩ, limiting the scope of simplification for the electronics. (iv) The arrays of PEGS also face a limitation in miniaturization due to the inherently larger size of paper-based structures compared with silicon more sophisticated, microfabricated devices. Nevertheless, given the wearable, standardized form factor (face mask) of our approach, further miniaturization would not substantially improve use or costs.

Using the PEGS array, we were clearly able to detect the consumption of licorice candy that contains ammonium chloride by eight healthy human subjects. We were, however, not able to detect the amount of candy (dose, from ¼ to ½) in a statistically significant fashion, although clearly the averages were visibly different. We believe that the way the candy is consumed (chewed vs. slowly sucked) impacts the measurement results of the exhaled analyte (NH_3_). The human experiments can be improved by more standardization of the testing protocols, and consumption of licorice can be a simple model to simulate diseased states similar to CKD or AKI. The performance of the sensor array can also be further improved in two different ways: (i) by performing measurements for a longer time for detecting low levels of analyte or (ii) by increasing the pH of the oral cavity, which would in turn improve the release of gaseous NH_3_.

Although, in this work, we primarily focused on the use of the PEGS array for detecting NH_3_ in human breath, its potential applications extend far beyond the topic described in this study. PEGS array can be utilized in various fields, such as the chemical industry for monitoring hazardous gases, medical diagnostics, agriculture, and environmental monitoring (26, 49–51). Because our approach uses a mobile device for data collection, the data produced by the PEGS array can be easily processed and stored on the cloud, enabling remote access by healthcare professionals, which is especially important in low- or middle-income countries with limited access to care. If mobile operation is not required, a simple LCD can also be implemented into our design to provide information at the point of care without connectivity. In the future, we will validate the application of PEGS arrays in clinical experiments to measure the levels of exhaled NH_3_ in patients suffering from AKI and CKD to monitor BUN levels noninvasively.

## Materials and methods

### Wearable respiratory monitor

For field testing, we developed a wearable respiratory monitor easily attached to a commercially available disposable medical face mask (Fig. 1E). The respiratory monitor for human testing consists of a medical face mask, plastic housing for the PEGS array (sensor chamber), and electronics (Fig. S3). The sensor chamber contains six PEGSs: three U-PEGSs and three A-PEGSs. The electronics chamber provides the electronic circuit to read, process, and transmit the sensor data. We designed the read-out component in-house and used Bluetooth low energy (BLE) to read and control the device with a smartphone (see SI-P3, Figs. S4 and S5). Human test subjects are asked to wear the whole setup and breathe normally through the mouth for 15 min. The exhaled breath passes the PEGS array and leaves through holes at the bottom of the sensor chamber.

Prior to conducting the study, a detailed risk assessment was performed according to the guidelines provided by Imperial College London. The risk assessment and standard operating procedure were reviewed and approved by the Departmental Safety Officer. As the study involved a wearable noninvasive device with minimal risk, it did not require formal review by an ethics panel. All human subjects provided informed consent for the sensor testing experiments, which were conducted exclusively on healthy subjects.

### Experimental setup for breathing test

We conducted four experiments in the following two different environments: in the first, a laboratory environment, to characterize the PEGS arrays, we designed a test chamber with known RH, flow rate, and gas concentration (see SI-P2). We can control the test environment and its parameters with three different gas lines: humid compressed air, which we humidified by passing it over the headspace of a DI water container; dry compressed air; and the target gas (i.e. NH_3_ or CO_2_) in air. In the second, we monitored healthy individuals with our wearable respiratory monitor (Fig. 1C).

Our experiments consisted of two characterization experiments in the sensor chamber and two tests on human subjects: in the first experiment, we exposed the PEGS array in controlled RH and flow rate to the target gas (NH_3_ and CO_2_) in our sensor chamber. We applied simulated breath (see below) with a tidal volume of ca. 270 mL and a breathing rate of 6 breaths/min. Both tidal volume and breathing rate are smaller than in average humans (tidal volume: 7 mL/kg, breathing rate: 10–20 breaths/min) (52) and are optimized for our system. We checked the PEGS array response to different concentrations of NH_3_ from 0.05 to 5 ppm in simulated exhalation. With our wearable respiratory monitor, we measured the NH_3_ content in the breath of healthy individuals before and after eating salty licorice (*Malaco Salmiak Balk Sweet* from Scandinavian Candy & Sweets). This Scandinavian candy contains a quantity (ca. 4–8%) of ammonium chloride (NH_4_Cl). While sucking on the candy, NH_3_ forms in the saliva and mixes into the exhaled breath through the mouth.

### Simulated human breathing

When breathing, humans do not use the full capacity of the lungs. If no extra effort is applied, the air exhaled while breathing is typically called tidal volume. An average human has a tidal volume of ca. 7 mL/kg and breathes between 10 and 20 times/min (52).

To simulate human breathing, we programmed mass flow controllers (type GM50A from MKS) in our test setup. The process involves a humidified air flow (>90% RH, 2,000 mL/min) passing over the sensors for 8 s, representing exhalation. This is followed by a 4-s airflow with RH similar to the room (∼50% RH, 2,000 mL/min), representing inhalation. We found that these timings closely matched the patterns of human breathing. The simulation, however, is not perfect and has the following shortcomings: (i) The exhaled volume is twice the inhaled volume. (ii) The tidal volume in this setup is ∼270 mL, half of the average human tidal volume (ca. 500 mL). (iii) The simulation completes six cycles per minute, slower than the average 8–12 breaths/min in humans. (iv) The temperature is constant, unlike the temperature difference between body and room temperature in human respiration. (v) We do not add CO_2_ to the exhalation, using the same cylinder of compressed air for both processes. Despite the mentioned shortcomings, our test setup can simulate the general shape of a breathing curve over time. To further mimic human respiration, we added different NH_3_ concentrations (0.1–5 ppm) to the exhalation stream, simulating oral NH_3_ production in humans.

## Supplementary Material

pgaf116_Supplementary_Data

## Data Availability

All data generated in this study have been deposited in the Zenodo database https://doi.org/10.5281/zenodo.14977214.

## Preprints

This manuscript was posted on a preprint at: https://doi.org/10.1101/2024.07.16.603629.
