# Supplementary material for: Wearable face mask-attached disposable printed sensor arrays for point-of-need monitoring of alkaline gases in breath
Source: PNAS Nexus. 2025 Apr 15;4(4):pgaf116. doi: 10.1093/pnasnexus/pgaf116 (PMC12038690; doi:10.1093/pnasnexus/pgaf116)
Supplement: pgaf116_Supplementary_Data [file pgaf116_supplementary_data.docx]

**Supplementary Information** for

**Wearable facemask-attached disposable printed sensor arrays for point-of-need monitoring of alkaline gases in breath**

Giandrin Barandun^a,b,1^, Abdulkadir Sanli^a,1^, Chun Lin Yap^a^, Alexander Silva Pinto Collins^a^, Max Grell^a,b^, Michael Kasimatis^a,b^, Jeremy B. Levy^c^, Firat Güder^a,*^

1. Department of Bioengineering, Imperial College London, SW7 2AZ, United Kingdom
2. BlakBear Ltd., 185 Tower Bridge Rd, London SE1 2UF, United Kingdom
3. Department of Immunology and Inflammation, Hammersmith Hospital, Imperial College London, W12 0HS, United Kingdom

*To whom correspondence should be addressed: Email: [guder@ic.ac.uk](mailto:guder@ic.ac.uk)

^1^G.B. and A.S. contributed equally to this work and are co-first authors.

**Table of Contents**

SI-P1. Characterization of A-PEGS for different NH_3_ concentrations

SI-P2. Test chamber for PEGS characterization

SI-P3. Electronics and software

SI-P4. Comparable technologies in literature

**SI-P1. Characterization of A-PEGS for different NH_3_ concentrations**

**
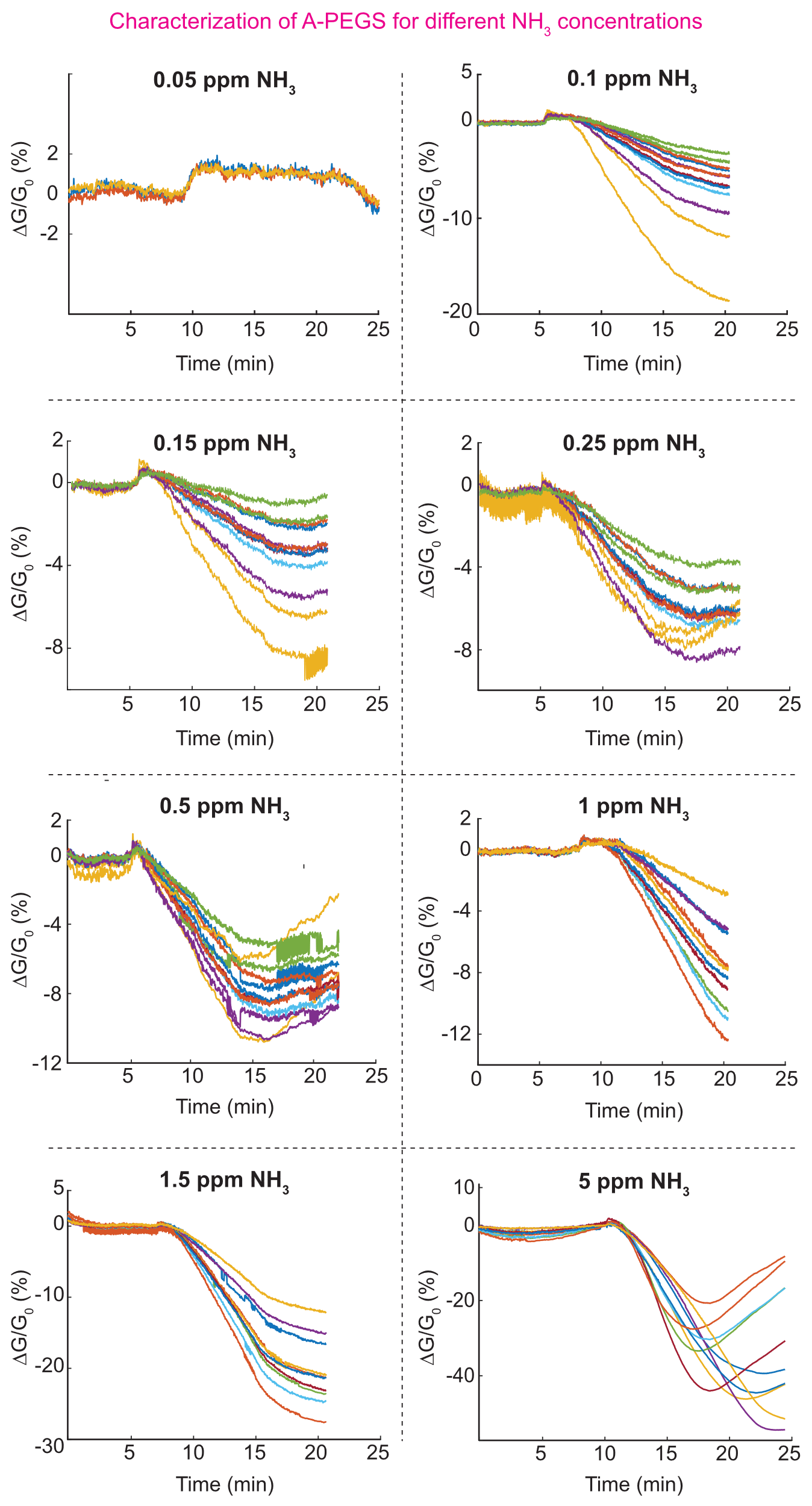
**

**Figure S1**: Characterization of A-PEGS. Change in conductance of A-PEGS for different amounts of NH₃, ranging from 0.05 ppm to 5 ppm. With increasing ammonia concentration, the slope also increases, indicating a linear correlation between NH₃ concentration and the conductance slope. The colors in the figure represent raw data collected from 8–12 subjects (n=8–12).

**Figure S1** shows the raw signal when exposed to ammonia in time (min^-1^) is shown for different concentrations of ammonia from 0.05 ppm to 5 ppm. For low concentrations (0.05 ppm to 0.25 ppm) the data shows higher standard deviation and less reliability on the linear correlation. We reach our lower limit of detection (LOD) at ca. 0.1 ppm for our test set-up. The current set-up, however, is limited in terms of mixing, flow rate and gas concentration. For low concentrations of ammonia, we mix high flow rates of compressed air (2000 ml/min) with very low flow rates of ammonia (10 ml/min). If the gas mixture is not perfectly homogeneous the target gas might show spatial differences in concentration (i.e. some areas of the chamber have higher concentrations whereas other areas are not reached at all. This can explain the higher errors and difficulties in detecting.

**SI-P2. Test chamber for PEGS characterization**

Three mass flow controllers (MFCs) (type GM50A from Bronkhorst UK Ltd) are programmed to adjust the flow rates of the three streams to keep the parameters in the test chamber at certain levels and can be controlled from a computer (**Figure S2**). The two lines of carrier gas are used to reach a precise RH level in the supply stream. This stream is mixed with the target gas and supplied into the test chamber. The test chamber is a polytetrafluoroethylene (PTFE) box (120×40×60 mm^3^) with two inlets at the top and one inlet on each small side to provide an evenly distributed supply of gas.


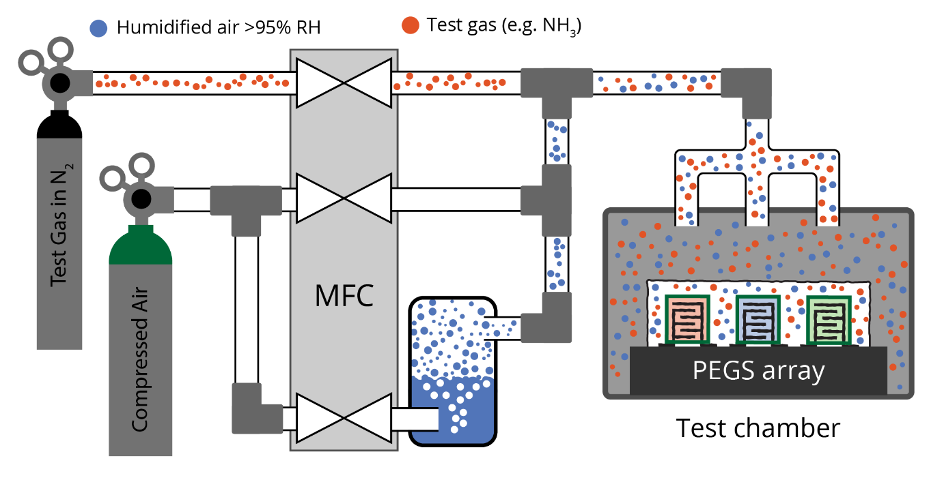


**Figure S2**: Test chamber set-up for the sensor characterization experiment. The carrier gas (compressed air) can either be used dry or humidified. To humidify the carrier gas, it bubbles through DI water (blue circles). The test gas (red circles) is mixed with the dry and humidified carrier gas. The mixing ratio is controllable using MFCs to achieve the desired RH and test gas concentration inside the test chamber containing the sensors.

With this set-up, we can test for a gas over a wide range of concentrations at different RH levels. For example, for ammonia we have a range of 1% to 100 ppb (part per billion). The flow rate of each line was adjusted with MFCs with a total flow rate of 2000 mL/min reaching the test chamber. The current set up, however, is limited in terms of mixing, flow rate and gas concentration. For low concentrations of ammonia, we mix high flow rates of compressed air (2000 ml/min) with very low flow rates of ammonia (10 ml/min). If the mixing is not perfect the target gas will reach areas of the chamber quicker or not reach other areas at all. This can explain the higher errors and difficulties in detecting.

We preferred intersurgical face masks available at EcoLite™ due to their widespread availability and affordability. We believe, however, that other brands could also serve the purpose effectively. Furthermore, we developed a 3D printed polylactic acid (PLA) housing for electronics to enhance the functionality of the masks.

**SI-P3. Electronics and software**


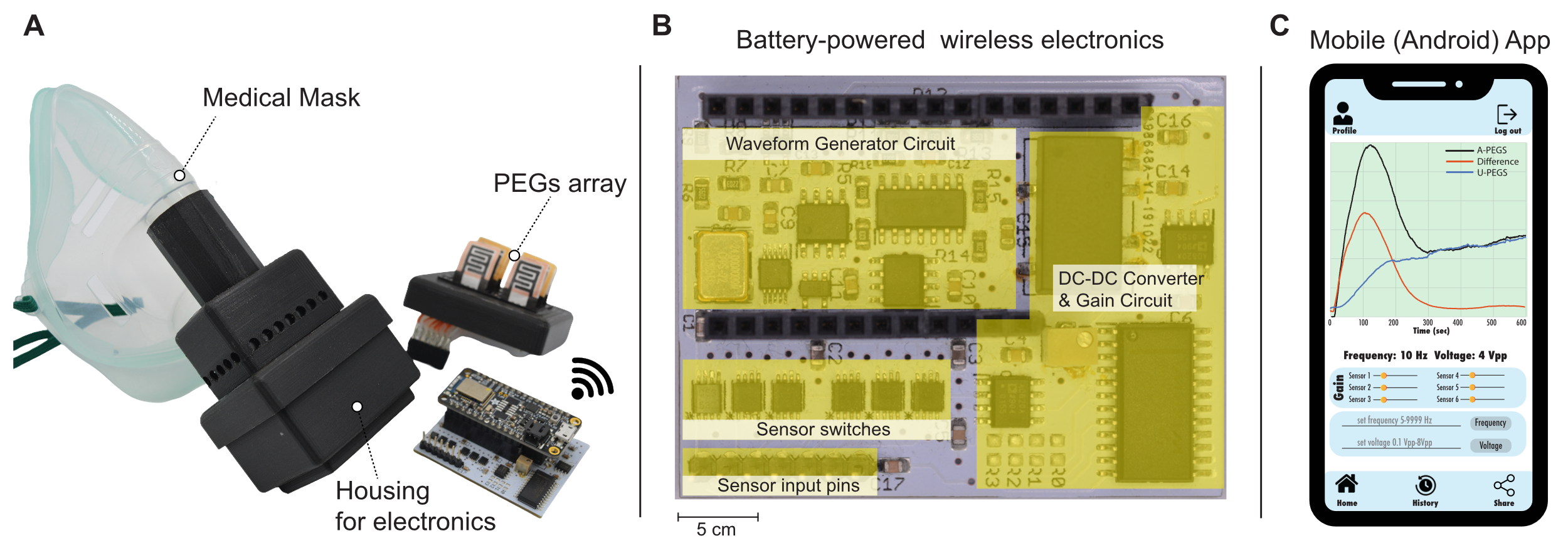


**Figure S3**: **Integrated system overview**. (**A**) Exploded view of the mask housing PEGS array and electronics for measurements. (**B**) Photograph of battery-powered wireless electronics for sensor data acquisition. (**C**) Image of the mobile (Android) app displaying and analyzing individual PEGS array readings, enabling comprehensive data visualization and analysis. The Android app was designed and implemented in the Android Studio development environment using Java and XML programming languages. We used an android phone (model Huawei P30 Lite) for our testing.

**Figure S3** provides an integrated system overview. In (**A**), an exploded view illustrates the medical mask housing the PEGS array and associated electronics for measurements. We preferred intersurgical face masks available at EcoLite™ due to their widespread availability and affordability. We believe, however, that other brands could also serve the purpose effectively. Furthermore, we developed a 3D printed polylactic acid (PLA) housing for electronics to enhance the functionality of the masks. Figure S3B shows a photograph of the battery-powered wireless electronics used for sensor data acquisition. **Figure S3C** displays an image of the mobile Android app, which visualizes and analyzes individual PEGS array readings for comprehensive data analysis. The app was developed in the Android Studio environment using Java and XML programming languages, and we used a Huawei P30 Lite phone for testing.


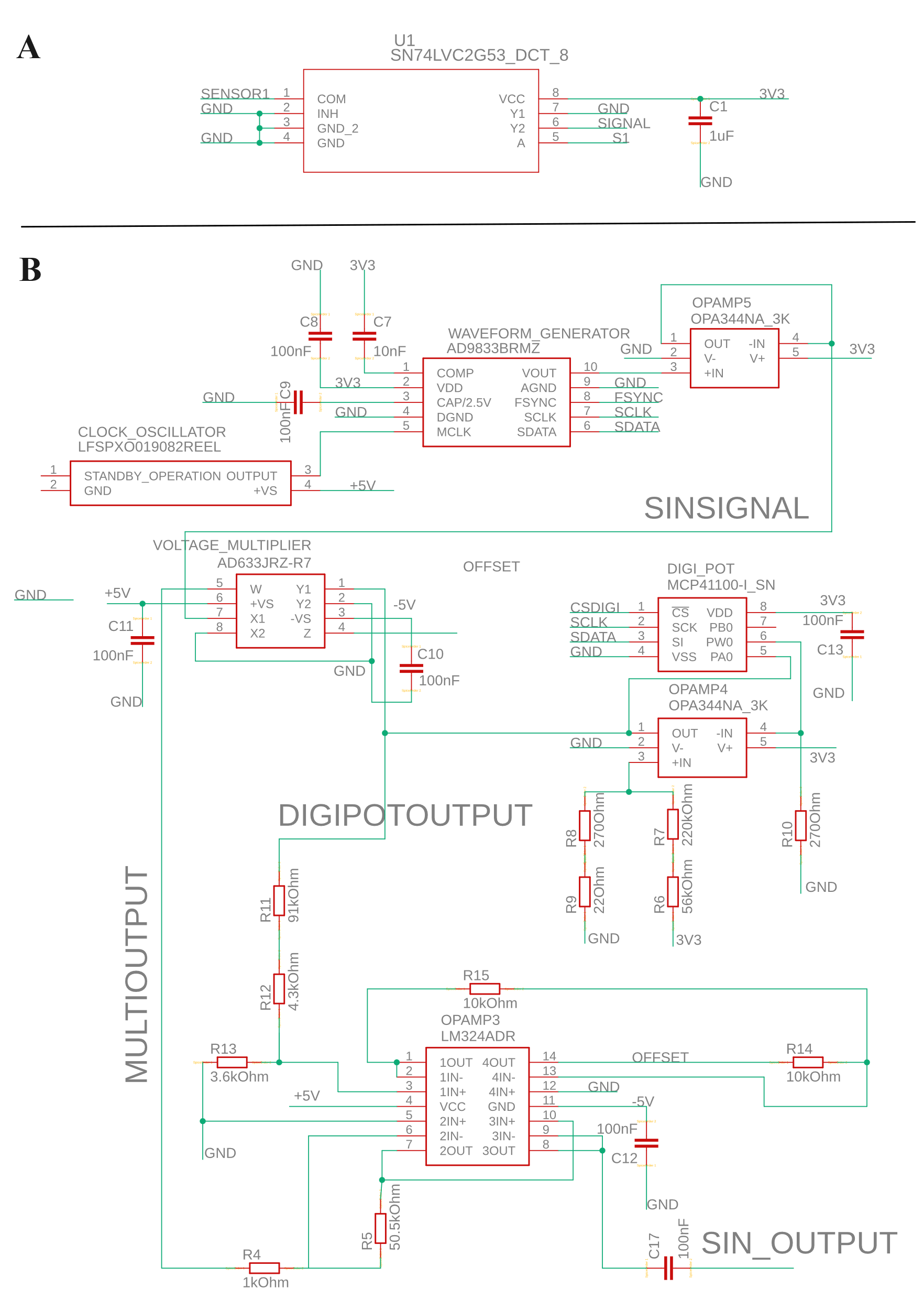


**Figure S4: (A)** Analog switch circuit for PEGS, **(B)** This circuit creates the sinusoidal wave to measure impedance in the PEGS array. A 24MHz clock oscillator clocks a waveform generator (AD9833) which is fed into a voltage follower (OPAMP5) to create the base signal ‘SINSIGNAL’ in a frequency between 10Hz-10kHz. The DC offset of the sinusoidal signal is then removed using a voltage multiplier (AD633) and amplified in a transimpedance amplifier configuration using an adjustable gain (digital potentiometer, MCP41100) to reach the desired voltage range (max. ca. +/- 4.2V). The final signal ‘SIN_OUTPUT’ is the input to the PEGS.

**
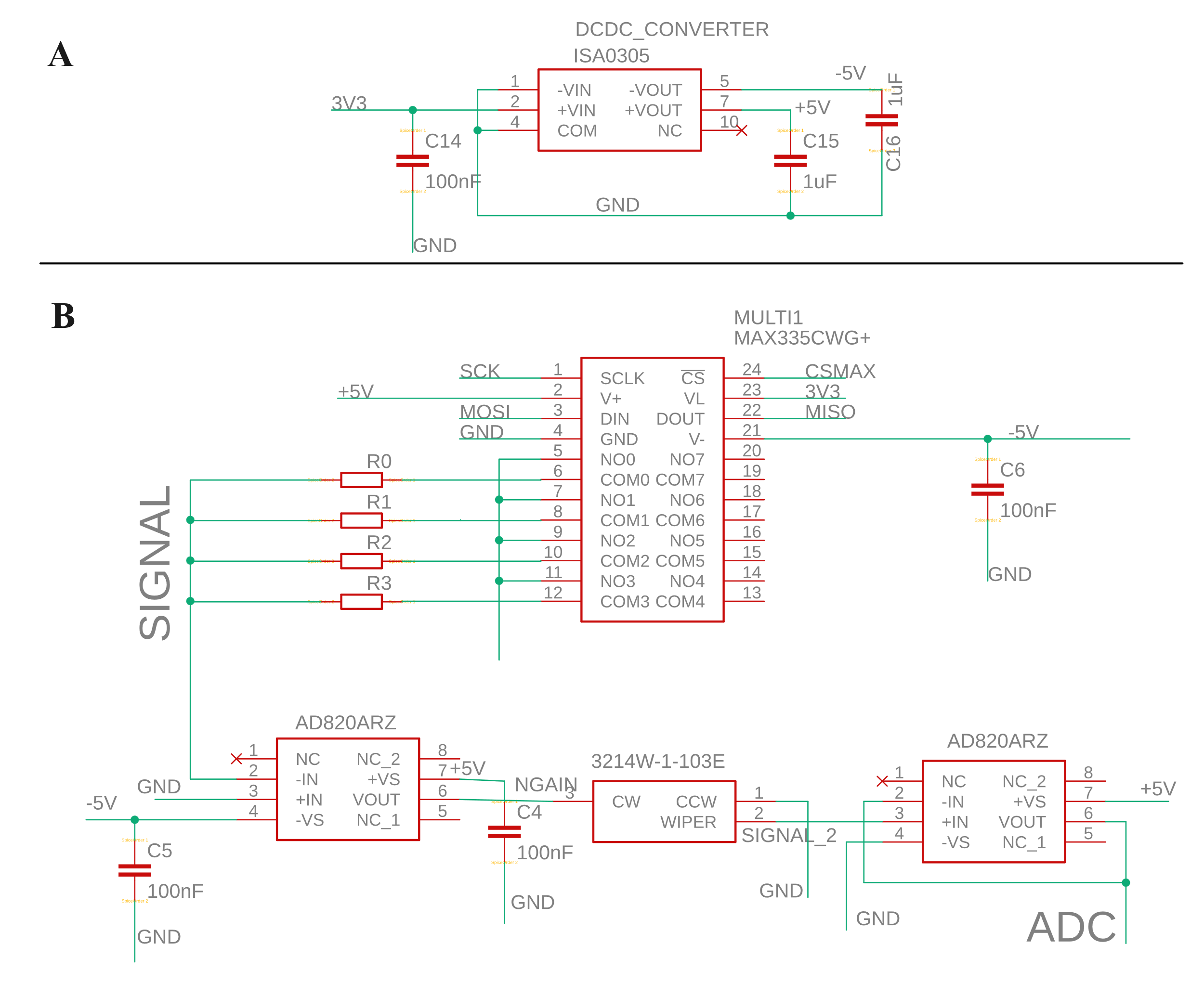
**

**Figure S5**: (**A**) A DC-DC converter is used to extend the 3.3V from a standard Arduino to -5 / +5 V to achieve proper AC sinusoidal waveforms (see Figure S4.A). **(B)** This circuit takes the sensor signal as an input, amplifies it, and feeds the signal into the Arduino ADC. The sensor input is ‘SIN_OUTPUT from Figure S4 and the output from the sensor (‘SIGNAL’) is amplified using a transimpedance amplifier configuration. A multiplexer (MAX335) chooses the correct gain resistor (50M, 10M, 1M, 100k) and a potentiometer (3214W) is used to downscale the 4Vpp signal range to 3.3V to be read by the Arduino ADC.

To measure the response to target gases, a sinusoidal excitation with an amplitude of 4 Vp-p at 10 Hz was applied, and conductance (G) was determined through Ohm’s law, enabling the calculation of ΔG/G_0_ to assess the sensor's reaction to the gas. For the impedance measurements, we followed the methodology outlined in our previous paper ^1^ (see specifically Figure 3). This choice was based on considerations of the Debye-Falkenhagen effect, and the capacitive charging behavior observed at different frequencies.

**Table S1**: A detailed breakdown of the bill of materials for producing a single PEGS in USD. The total estimated cost is approximately US $0.02 per sensor.

| **Component** | **Details** | **Price* (USD)** | |
| --- | --- | --- | --- |
| Paper (substrate) | \|  \| \| --- \|  \| Whatman™ chromatography paper (pack of 500 sheets costing £770; each sheet yields ~418 sensors) \| \| --- \| | | 0.0047 |
| Conductive ink | Carbon ink (e.g., $38 for 50 mL; ~0.015 mL used per sensor) | | 0.0114 |
| Wax | Wax for hydrophobic barriers (e.g., $25 for 1 kg; ~0.01 g per sensor) | | 0.002 |
| **TOTAL (USD)** |  | | **~0.02** |

* For the fabrication of the paper sensors, we mixed conductive carbon ink (No. C2130925D1, GWENT Group) in a ratio of 55/45 wt % with a diluent (No. S60118D3, GWENT Group). To fabricate the electrodes, we used a cutter plotter (GRAPHTEC, Model CE6000-40) and a ballpoint pen (Sakura Gelly Roll METALLIC). The substrate was Whatman™ chromatography paper (Sigma Aldrich Product no WHA1001918). Prices per unit are converted from GBP using the prevailing exchange rate, subject to change.

**Table S2**: Prices of components (in USD) used for the fabrication of the electronic circuits and the corresponding equivalent platform using Bluetooth for data communication.

| **Component** | **Price* (USD)** |
| --- | --- |
| Bluefruit LE development board (Adafruit Feather M0) | 24.95 |
| Waveform generator (AD9833) | 11.87 |
| Analog voltage multiplier (AD633JRZ-R7) | [12.44](https://www.mouser.co.uk/ProductDetail/Analog-Devices/AD633JRZ-R7?qs=NmRFExCfTkGRW5FCzjwFTA%3D%3D) |
| Analog switches (MAX335) | 7.29 |
| DC-DC Converter (ISA0305) | 3.70 |
| Precision operational amplifier (AD820ARZ) | 6.38 |
| Resistors/ Capacitors/ Potentiometer | 2.15 |
| PCB manufacturing | 4.69 |
| PLA housing | 0.67 |
| Li-Po Battery charger circuit | 1.49 |
| **TOTAL (USD)** | **75.63** |

* Prices per unit are based on ordering 10 units and are converted from GBP using the prevailing exchange rate, subject to change. All components were purchased from Mouser Electronics.

**SI-P4. Comparable technologies in literature**

**Table S3.** Overview of PANI in gas sensing. A selection of PANI-based materials is used as a sensitive layer for ammonia detection. Abbreviations: 1) PEO: poly [ethylene oxide]; 2) MWCNT: multi-walled carbon nanotubes.

| **PANI Material** | **LOD** | **Response Time** | **Reversibility** | **Year ^[Ref]^** |
| --- | --- | --- | --- | --- |
| PANI-PEO ^1)^ | 0.5 ppm | 75s | yes | 2004^2^ |
| PANI-SWCNT^2)^ | 0.05 | Order of mins | yes | 2006^3^ |
| PANI-AG | <5ppm | 10-30min | yes | 2009^4^ |
| PANI-TIO_2_-AU | 1 ppm | 52s-122s | yes | 2017^5^ |
| PANI-MWCNT-AG | 2 ppm | 5s | yes | 2017^6^ |

**References**

1. Barandun, G. *et al.* Cellulose Fibers Enable Near-Zero-Cost Electrical Sensing of Water-Soluble Gases. *ACS Sens* **4**, 1662–1669 (2019).

2. Liu, H., Kameoka, J., Czaplewski, D. A. & Craighead, H. G. Polymeric Nanowire Chemical Sensor. *Nano Lett* **4**, 671–675 (2004).

3. Zhang, T., Nix, M. B., Yoo, B., Deshusses, M. A. & Myung, N. V. Electrochemically Functionalized Single‐Walled Carbon Nanotube Gas Sensor. *Electroanalysis* **18**, 1153–1158 (2006).

4. Gao, Y. *et al.* Silver/Polyaniline Composite Nanotubes: One-Step Synthesis and Electrocatalytic Activity for Neurotransmitter Dopamine. *The Journal of Physical Chemistry C* **113**, 15175–15181 (2009).

5. Liu, C. *et al.* Enhanced ammonia-sensing properties of PANI-TiO2-Au ternary self-assembly nanocomposite thin film at room temperature. *Sens Actuators B Chem* **246**, 85–95 (2017).

6. Abdulla, S., Ponnuvelu, D. V. & Pullithadathil, B. Rapid, Trace‐Level Ammonia Gas Sensor Based on Surface‐Engineered Ag Nanoclusters@Polyaniline/Multiwalled Carbon Nanotubes and Insights into Their Mechanistic Pathways. *ChemistrySelect* **2**, 4277–4289 (2017).
